# Impact of birth season on the years of life lost from respiratory diseases in the elderly related to ambient PM_2.5_ exposure in Ningbo, China

**DOI:** 10.1186/s12199-021-00994-6

**Published:** 2021-07-17

**Authors:** Teng Yang, Tianfeng He, Jing Huang, Guoxing Li

**Affiliations:** 1 Department of Occupational and Environmental Health Sciences, School of Public Health, Peking University, 38 Xueyuan Road, Beijing, 100191 China; 2 Ningbo Municipal Center for Disease Control and Prevention, Ningbo, 315010 China

**Keywords:** Birth season, Fine particle, Respiratory diseases, Years of life lost

## Abstract

**Background:**

Ambient fine particle (PM_2.5_) pollution is an important public health problem in China. Short-term ambient PM_2.5_ exposure is associated with increased mortality of respiratory diseases. However, few evidence was available on the effect of exposure to ambient PM_2.5_ on the years of life lost (YLL) from respiratory diseases in the elderly. Furthermore, birth season which is frequently applied as a proxy for environmental exposure in early life may influence the health outcome in the later life. Nevertheless, the modification effect of birth season on the relationship of PM_2.5_ exposure and respiratory health need to be explored.

**Methods:**

A time-stratified case-crossover design was used to analyze YLL from respiratory diseases in the elderly related to ambient PM_2.5_ exposure between 2013 and 2016 in Ningbo, China. The modification effect of birth season was explored by subgroup comparisons between different birth seasons.

**Results:**

Each 10 μg/m^3^ increase in daily ambient PM_2.5_ was associated with an increment of 1.61 (95% CI 0.12, 3.10) years in YLL from respiratory diseases in the elderly population. Individuals who were born in winter had significantly higher YLL from respiratory diseases associated with ambient PM_2.5_ exposure than those who were born in other seasons.

**Conclusions:**

Birth season which reflects the early-life PM_2.5_ exposure level that may influence the lung development has a potential effect on the disease burden of respiratory diseases related to ambient PM_2.5_ exposure in later life. The results would provide theoretical basis to protect vulnerable population defined by birth season when exploring the adverse effects of ambient PM_2.5_ in the respiratory health.

**Supplementary Information:**

The online version contains supplementary material available at 10.1186/s12199-021-00994-6.

## Background

Ambient fine particular matter (PM_2.5_) pollution has become an important environmental and public health problem and draws great concerns worldwide for its contribution to global disease burden [1]. In the past decades, China has experienced rapid economic growth, urbanization, and industrialization that cause severe PM_2.5_ pollution, which makes it one of the most ambient PM_2.5_-polluted countries [2]. Even though China issued the Air Pollution Prevention and Control Action Plan in 2013 to reduce the air pollution level, the annual average PM_2.5_ concentration in 2017 (47.0 μg/m^3^) was still higher than the World Health Organization (WHO) guideline level (10 μg/m^3^, 2005) [3].

Respiratory diseases mainly including acute upper respiratory infections, influenza and pneumonia, chronic obstructive pulmonary disease (COPD), and asthma are common diseases and pose a serious threat to health. For instance, chronic respiratory diseases are among the primary causes of morbidity and mortality worldwide [4]. PM_2.5_ could go through the respiratory tract and accumulate in the lung parenchyma which cause respiratory diseases such as acute lower respiratory infections and chronic obstructive pulmonary disease [5].

Epidemiological studies have demonstrated that exposure to short-time ambient PM_2.5_ is associated with increased mortality of respiratory diseases, and each 10 μg/m^3^ increment in ambient PM_2.5_ level is significantly associated with 0.5~2.0% increased risk of respiratory mortality [6, 7]. However, there are only a few studies that explored the association between ambient PM_2.5_ exposure and respiratory mortality in the elderly, and the results are conflict [8, 9]. Under the background of population aging, studies in the elderly have important implications as aging has become a significant social and public health challenge. In addition, studies exploring the effect of ambient PM_2.5_ exposure on the years of life lost (YLL) from respiratory diseases are scarce. Compared with mortality, the disease burden indicator of YLL is more comprehensive for bringing in life expectancy at death into consideration [10].

Although the modifications of traditional demographic characteristics in the health effects of PM_2.5_ exposure are frequently analyzed, the impact of birth season is rarely taken into consideration. However, birth season is frequently applied as a proxy for a wide range of environmental and other factors exposed in prenatal and early postnatal life such as air pollution, sun exposure, and nutritional status [11, 12]. These factors in the early life may influence the health outcome in later life. Previous studies have indicated the associations between birth season and mortality [13, 14]. Thus, whether birth season plays a role on YLL from respiratory diseases in the elderly related to ambient PM_2.5_ exposure remains an interesting topic to be explored.

This study aims to assess the impact of exposure to ambient PM_2.5_ on the disease burden of respiratory diseases by the indicator of YLL in the elderly in Ningbo, China, and to explore whether the modification effect of birth season exists. The results will provide scientific evidence for ambient PM_2.5_ control and susceptible population protection.

## Methods

### Study site and data collection

Ningbo city is the study site, which is seated in the Yangtze River Delta in southern China. As a typical Jiangnan water town and seaport city, chemical, textile, and machinery industry are the three pillar industries of Ningbo. It has a subtropical monsoon climate and the four seasons are distinct [15]. Meanwhile, 6.27% of Ningbo population are ≥ 75 years with a total population of 5.83 million during the study period [16].

Daily concentrations of PM_2.5_ and other three gaseous pollutants including 8-h maximum ozone, nitrogen dioxide (NO_2_), and sulfur dioxide (SO_2_) between 1 January 2013 and 31 December 2016 were obtained from the Environment Monitoring Center of Ningbo. The monitoring data of air pollutants were collected from 8 fixed monitoring sites in Ningbo, which covered both urban and suburban areas. Thus, the air pollution levels of whole Ningbo city were substituted by the average values of air pollutants concentrations from these monitoring sites. Daily meteorological data consisting of temperature and relative humidity were obtained from Ningbo Meteorological Bureau. The missing data percentages of air pollutants and meteorological variables were no more than 1%, and they were substituted by the daily mean value when cleaning data.

Daily mortality data in the elderly population from the Ningbo Municipal Center for Disease Control and Prevention were collected. Previous definition of elderly individuals was referred that those whose age were not lower than 75 years [17]. Those whose primary cause of death was respiratory diseases according to the International Classification of Diseases, 10th reversion, were selected. Codes J00–J99 were applied to count daily mortality from respiratory disease in the elderly population [18].

Registered residents were demanded strictly, and necessary information such as gender, birthdate, and age were all included in the database. According to the definition of the China Meteorological Administration, the continuous 12 months from March this year to February next year were divided into four seasons with every 3 months as a phase, such as spring representing March, April, and May and winter including December, January, and February. YLL from respiratory diseases in the elderly population was calculated by matching the age of each death from respiratory diseases to the WHO standard life table (Table S1). Daily YLL was the collection of YLL from the death on the same day. With this context, birth season was used to stratify the deaths and daily YLL from respiratory diseases in the elderly population.

### Statistical analysis

The correlations of PM_2.5_ with other gaseous pollutants and metrological conditions were analyzed by the Spearman correlation function. To explore the impact of ambient PM_2.5_ exposure on YLL from respiratory diseases, a time-stratified case-crossover design which was one type of time series analysis was applied in the study. A generalized additive model (GAM) was used to estimate the associations. Based on previous studies [19, 20], the link function was the identity function, and regression model with penalized splines was constructed as follows:

$$ {YLL}_t=\alpha +\sum \limits_{i=1}^q\beta i(Xi)+\sum \limits_{j=1}^p fj\left( Cj, df\right)+{W}_t(week)+ Strata $$

*YLL*_*t*_ refers to the daily YLL from respiratory diseases in the elderly population at day t. α is the intercept. *Xi* represents the daily mean concentration of ambient PM_2.5_, while *βi* represents the coefficient of YLL in relation to a unit rise in ambient PM_2.5_. *fj* represents the penalized spline function. Confounding factors consisting of daily temperature and daily relative humidity are controlled in *Cj*, and their *df* were set to 3 [21]. Considering the lag days and lag structure of temperature and relative humidity, the 14-day moving average of temperature [22] and the average value of current day of relative humidity [23] were used in our models. The day of week effect is reflected in *W*_*t*_(*week*). To control the long-term trends, *S*trata which is a categorical variable of the year and calendar month was used in the models [22]. A single lag day (from lag0 to lag7) and the moving average over the lag days (from mv01 to mv07) were used to estimate the potentially delayed effects and cumulative associations of ambient PM_2.5_ exposure on the elderly population, respectively. The change in daily YLL from respiratory diseases with each 10 μg/m^3^ increase in ambient PM_2.5_ was used to reflect the effect.

To explore the modification effect of birth season, subgroup comparisons between different birth seasons were applied in the analysis. YLL from respiratory diseases with an increase of 10 μg/m^3^ in ambient PM_2.5_ for those born in whole year and four seasons was calculated. Furthermore, statistical test was used to evaluate the difference of birth seasons as shown in the following:

$$ \left({\upbeta}_1-{\upbeta}_2\right)\pm 1.96\sqrt{{SE_1}^2+{SE_2}^2} $$

In the equation, β_1_ and β_2_ are the estimates for the compared two groups (e.g., spring and winter), and *SE*_*1*_ and *SE*_*2*_ are their corresponding standard errors.

Besides the single-pollutant model exploring the primary association between ambient PM_2.5_ and YLL from respiratory diseases, the two-pollutant model was also constructed to examine whether the association was robust when adding ozone, NO_2_, or SO_2_ to the model. In addition, the exposure-response curves in four birth seasons were plotted using penalized spline smoothing function to explore the relationship between ambient PM_2.5_ exposure and YLL from respiratory diseases. Furthermore, when calculating the excess risk of death from respiratory diseases in the elderly population associated with per 10 μg/m^3^ PM_2.5_ increase, the linear term was applied on behalf of the penalized spline. Relative risk (RR) was estimated by the coefficient *β*, and the excess risk was calculated by the formula (RR − 1) × 100%.

The Institutional Review Board of Ningbo Municipal Center for Disease Control and Prevention approved our study (No. IRB 201603). All analyses were performed by R software (version3.1.2, http://www.R-project.org/). Two-sided *p* < 0.05 was defined as the criterion for statistical significance.

## Results

**Fig. 1:**
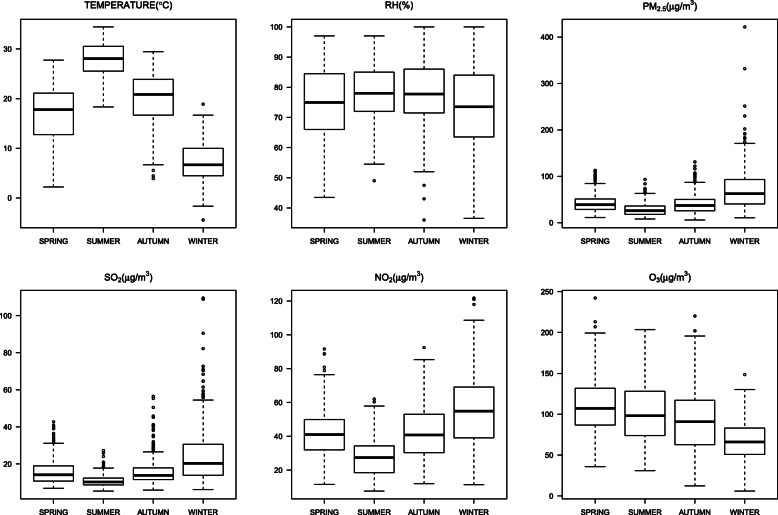
Box plots of seasonal meteorological conditions, air pollutants in Ningbo, China, from 2013 to 2016

The mean PM_2.5_ concentration was 45.5 (SD 32.0, range 5.9, 421.7) μg/m^3^ from 2013 to 2016 in Ningbo, China [23]. The concentration of PM_2.5_ was relatively highest in winter, while the concentration of ozone was lowest in winter (Fig. 1). The Spearman correlation coefficients of PM_2.5_ with other air pollutants and meteorological factors are shown in the supplementary materials (Table S2). PM_2.5_ was positively associated with NO_2_ and SO_2_, while negatively in relation to ozone, temperature, and relative humidity.

From 2013 to 2016, the total number of deaths and YLL from the respiratory diseases were 18,989 and 202,969.9 years, respectively. The mean of daily death counts and corresponding YLL from respiratory diseases are shown in Table 1. Compared with those born in autumn and winter, relatively fewer daily death counts and YLL were found in those born in spring and summer.

**Table 1:** Daily number of deaths and YLL in the elderly population born in different seasons

Variables	Mean ± SD	Minimum	P25	Median	P75	Maximum
**Daily death counts**						
Whole year	13.0 ± 5.4	2.0	9.0	12.0	16.0	32.0
Spring-born	2.5 ± 1.7	0.0	1.0	2.0	4.0	10.0
Summer-born	2.6 ± 1.8	0.0	1.0	2.0	4.0	10.0
Autumn-born	3.9 ± 2.3	0.0	2.0	4.0	5.0	15.0
Winter-born	4.0 ± 2.3	0.0	2.0	4.0	5.0	14.0
**Daily years of life lost (years)**						
Whole year	138.9 ± 59.2	12.7	96.3	128.0	170.8	361.1
Spring-born	26.9 ± 19.2	0.0	12.2	24.4	37.8	104.8
Summer-born	28.4 ± 20.7	0.0	12.9	25.0	39.4	130.4
Autumn-born	41.3 ± 25.4	0.0	23.0	38.2	56.2	157.1
Winter-born	42.3 ± 25.6	0.0	22.9	38.3	58.9	149.9

### Association of ambient PM_2.5_ with YLL from respiratory diseases

**Fig. 2:**
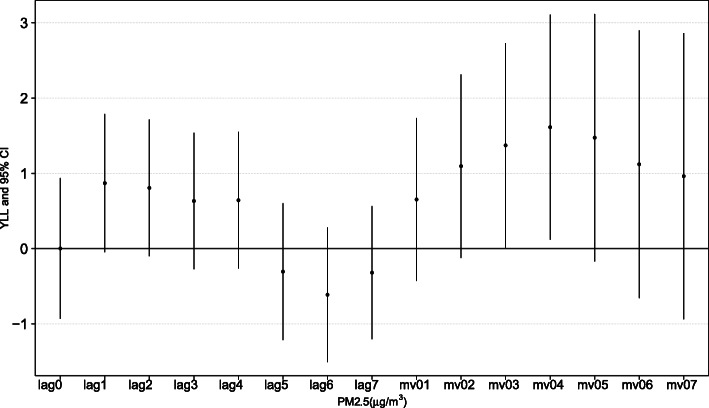
Estimated changes in YLL with each 10 μg/m^3^ increase in PM_2.5_ on different lag days

Corresponding changes with 95% confidence in YLL from respiratory diseases with each 10 μg/m^3^ rise in ambient PM_2.5_ on different lag days in the elderly individuals are shown in Fig. 2. The estimated changes in YLL from respiratory diseases were positive from lag1 to lag4, while the trend attenuated at lag5. There was a peak at 4-day moving average (mv04) concentration which implicated the strongest cumulative effect, with an increment in YLL from respiratory diseases of 1.61 (95% CI 0.12, 3.10) years in the elderly population in relation to each 10 μg/m^3^ increase in daily ambient PM_2.5_. Mv04 was applied to the main statistical analysis for its strongest effect.

### Modification of birth season

Table 2 shows the associations between per 10 μg/m^3^ increase in ambient PM_2.5_ and YLL from respiratory diseases in the elderly population born in different seasons. When the single-pollutant model was stratified by birth season, YLL from respiratory diseases associated with per 10 μg/m^3^ increase of ambient PM_2.5_ exposure was significantly higher in the elderly population born in winter in comparison with other seasons. To be specific, a significant increase in YLL from respiratory diseases of 1.54 (95% CI 0.75, 2.34) years associated with per 10 μg/m^3^ increase in ambient PM_2.5_ concentration was observed in the winter-born elderly population. There were no significant associations between YLL from respiratory diseases in the elderly born in other seasons.

**Table 2:** Associations between per 10 μg/m^3^ increase of ambient PM_2.5_ and YLL

Variables	Whole year (95%CI)	Spring-born (95%CI)	Summer-born (95%CI)	Autumn-born (95%CI)	Winter-born (95%CI)
**Single-pollutant model**	1.61 (0.12, 3.10)	− 0.14 (− 0.76, 0.48)^*^	0.34 (− 0.32,0.99) ^*^	− 0.13 (− 0.91,0.66) ^*^	1.54 (0.75, 2.34)
**Two-pollutant model**					
+NO_2_	0.83 (− 1.16,2.83)	− 0.58 (− 1.32, 0.15) ^*^	0.26 (− 0.62, 1.13) ^*^	− 0.29 (− 1.34,0.76) ^*^	1.76 (0.70, 2.82)
+SO_2_	0.47 (− 1.35, 2.29)	− 0.29 (− 1.05, 0.46) ^*^	− 0.15 (− 0.95,0.65) ^*^	− 0.46 (− 1.42,0.50) ^*^	1.38 (0.41, 2.35)
+O_3_	1.05 (− 0.57, 2.66)	− 0.41 (− 1.07, 0.26) ^*^	0.29 (− 0.42, 1.00) ^*^	− 0.24 (− 1.09, 0.62) ^*^	1.40 (0.54, 2.26)

Results are presented at mv04. The unit of the values is years. *p < 0.05 compared with the years of life lost in the winter-born elderly population

The results were robust when NO_2_, SO_2_, and ozone were considered in the two-pollutant model, respectively. For instance, when NO_2_ was added to the model, per 10 μg/m^3^ increase of ambient PM_2.5_ was associated with 1.76 years (95% CI 0.70, 2.82) increase in YLL from respiratory diseases in the elderly population born in winter, which was significantly higher compared with YLL estimated in other birth seasons (Table 2).

The excess risk of respiratory diseases mortality with ambient PM_2.5_ exposure presented similar trend, with higher risk in those born in winter as the results of YLL (Table S3). However, significant difference was not found in the summer-born population but in the spring-born and autumn-born population when compared with winter-born individuals in single-pollutant model.

### Exposure-response relationship

**Fig. 3:**
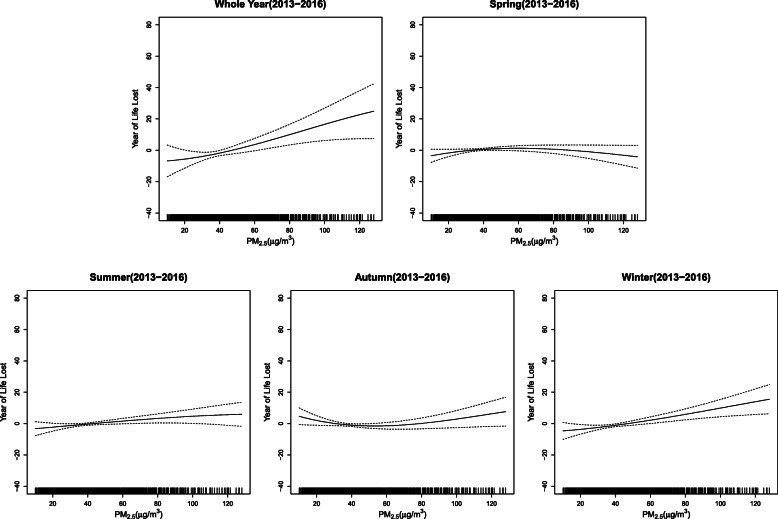
Exposure-response curves of PM_2.5_ and YLL in the elderly population born in different seasons

The exposure-response curves of ambient PM_2.5_ exposure and YLL from respiratory diseases showed various patterns for different birth seasons (Fig. 3). There was an approximate linear relation for PM_2.5_ exposure in the elderly individuals born in winter. The exposure-response curve was relatively steep for those born in winter compared with other three seasons and had the similar positive trend with the curve for the whole year.

## Discussion

In this study, YLL from respiratory diseases was estimated to explore the relationship of ambient PM_2.5_ exposure and the disease burden in the elderly population in Ningbo, China. Especially, a significant association between ambient PM_2.5_ exposure and YLL from respiratory diseases in the elderly individuals was found. Furthermore, birth season played a potential modification effect on the relationship that YLL from respiratory diseases related to ambient PM_2.5_ exposure was higher in the elderly who were born in winter than those born in other seasons.

Ambient PM_2.5_ pollution is a great challenge to public health, both in developed and developing countries [24, 25]. Short-term exposure to low-concentration PM_2.5_ is associated with increased mortality as well, which raises concerns about PM_2.5_ pollution [25]. In China, the concentration of PM_2.5_ is respectively lower in south areas than in north areas, and higher in winter compared with other three seasons [26]. The annual average concentration in our research site located in the Yangtze River Delta was lower than the mean concentration of whole China and showed the highest level in winter.

Our results showed that an increase of 10 μg/m^3^ in ambient PM_2.5_ was associated with 1.06% (95% CI 0.17%, 1.95%) increase in mortality of respiratory diseases, and this result was consistent with previous studies [6, 7]. Furthermore, relatively stronger effect of exposure to ambient PM_2.5_ on mortality of respiratory diseases in the elderly was observed in our study. Li et al. found that each 10 μg/m^3^ increment in PM_2.5_ was related to an increase of 0.51% (95% CI 0.30%, 0.73%) in respiratory mortality in all-ages [27]. Sui et al. found that each 10 μg/m^3^ in PM_2.5_ increased death risk of respiratory diseases by 0.90% (95% CI 0.23%, 1.57%) [8]. Compared with the former two studies in China, Ningbo showed relatively lower mean PM_2.5_ concentration (45.5 μg/m^3^ in Ningbo, 84.9 μg/m^3^ in Beijing, 66.18 μg/m^3^ in Hefei) and higher increased mortality risk. The reason may be that the elderly is more vulnerable to the effect of PM_2.5_ pollution on respiratory diseases [9].

To our knowledge, the effect of exposure to ambient PM_2.5_ on YLL from respiratory disease in the elderly was evaluated for the first time. Per 10 μg/m^3^ PM_2.5_ increase was significantly associated with an increase of 1.61 (95% CI 0.12, 3.10) years in YLL from respiratory diseases in the elderly. Li et al. found that each 10 μg/m^3^ increase in ambient PM_2.5_ was related to 0.98 (95% CI 0.42, 1.54) years increment in YLL from COPD in the elderly (≥ 75 years), and COPD is one of the major types of respiratory diseases [28]. Since the mean PM_2.5_ concentration of the two studies is similar (45.5 μg/m^3^ VS 49.58 μg/m^3^), it indicates that COPD may account for major percentages in YLL from respiratory diseases. This recommends us to explore the association of ambient PM_2.5_ with YLL from other respiratory diseases such as asthma, which is a common respiratory disease as well.

Birth season has been demonstrated to be a risk factor of population mortality in previous studies. Zhang et al. found that women born in spring and summer had a higher cardiovascular mortality than women born in the autumn [14]. Our results provided new insights that birth season may increase the disease burden of respiratory diseases in the elderly. As a unique birth season, winter had a potential modification effect on YLL from respiratory diseases related to ambient PM_2.5_ exposure. Similar effect has not been discovered in other research up to now.

Chronic respiratory diseases account for an essential part of respiratory diseases and were the third leading cause of death next to cardiovascular diseases and neoplasms in 2017 [4]. According to the Development Origins of Health and Disease, environmental factors work during the phase of developmental plasticity to change the susceptibility of the individuals to the noncommunicable diseases in later life [29]. Early life encompassing prenatal and early childhood has the potential to act as a window of sensitivity to ambient PM_2.5_ exposure, which increases the risk of chronic respiratory diseases [30].

Lung development lasts for a long time from utero to early adulthood, and the first year after birth is greatly important to substantial structural development of lung [31]. The developing lung is highly susceptible to ambient pollutants [32]. Gauderman et al. showed that exposure to PM_2.5_ was associated with delayed development of lung function in a prospective study [33]. In our study, the ambient PM_2.5_ concentration was highest in winter, followed by spring, in Ningbo, China. Compared with other seasons, people spend more time indoors in winter for the relatively lower temperature outdoors. Research found that ambient air was the major source of indoor PM_2.5_ in winter in the Yangtze River Delta [34]. Besides, increased indoor activity and decreased ventilation frequency contributed to high indoor PM_2.5_ level in winter. Thus, those born in winter are exposed to higher PM_2.5_ pollution level in their early life, and may induce irreversible damage to their lung development which influences their sensibility to respiratory diseases in later life.

Cold temperature is another possible risk factor of respiratory mortality in later life for those born in winter. Research found that cold weather contributed to the incidence of respiratory diseases [35]. On the one hand, low temperature is beneficial for bacteria to survive in water droplets [36]. Especially, newborns show evident differences to the adult in immune function, which increase their susceptibility to respiratory diseases [37]. On the other hand, cold weather and corresponding holidays in China limit accessibility to medical facilities and may delay the treat time. Adverse respiratory events in early life would be related to susceptibility to air pollution in later life.

This study has the following strengths. First, birth season indicates PM_2.5_ exposure level in early life may influence lung development in the later life. Nevertheless, the impact of birth season in the respiratory health effects associated with ambient PM_2.5_ is rarely explored. To the best of our knowledge, our study analyzed the potential modification effect of birth season on YLL from respiratory diseases related to ambient PM_2.5_ exposure for the first time. Second, the effects of PM_2.5_ on the respiratory health in the elderly were investigated in our study. With the continuous population aging trend, it is of great public importance to explore the risk factor of respiratory diseases in the elderly. Third, YLL was calculated to assess the disease burden resulting from respiratory diseases in our study. YLL comprises life expectancy at death and assigns different weights to deaths occurred at various ages [19]. Thus, YLL is more accurate for quantifying premature deaths than mortality and reflects the disease burden of PM_2.5_ exposure better. From the public health implication prospect, YLL is more informative for resource allocation and policy-making [21].

However, some limitations existed in our study. First, our study site was a single city located in South China and had relatively lower ambient PM_2.5_ concentration compared with other Chinses cities. Thus, the extrapolation of the results to other geographical areas should be taken with caution. Second, personal PM_2.5_ exposure was substituted by fixed monitoring data due to data availability. However, this would cause measurement bias that the effect may step toward null [38]. Third, the components of PM_2.5_ associated with respiratory diseases were not considered in our study. Considering the toxicity of different components of PM_2.5_ varied, it is meaningful to explore the effect of PM_2.5_ components on YLL from respiratory diseases in the elderly if data are available.

## Conclusions

This study suggests that exposure to ambient PM_2.5_ is the risk factor of disease burden from respiratory diseases in the elderly. Birth season reflects the exposure level of PM_2.5_ in early life, and those born in winter are more vulnerability to respiratory diseases in the elderly. The results indicate a potential risk factor for respiratory diseases in later life. This study provides theoretical basis for the update of air standards for PM_2.5_ and the protection of vulnerable populations and has important public health implications.

## Supplementary Information

**Additional file 1: Table S1.** WHO standard life table for years of life lost. **Table S2.** Spearman correlations between air pollutants and meteorological conditions in Ningbo, China, from 2013 to 2016. **Table S3.** Associations between per 10 μg/m^3^ increase in PM_2.5_ and excess risk of respiratory diseases mortality.

## Data Availability

The datasets used and/or analyzed during the current study are available from the corresponding author on reasonable request.

## Abbreviations

PM_2.5_: Fine particular matter
WHO: World Health Organization
COPD: Chronic obstructive pulmonary disease
YLL: Years of life lost
NO_2_: Nitrogen dioxide
SO_2_: Sulfur dioxide
lag4: Lag day 4
mv04: 4-day moving average
