# Supplementary material for: Impact of birth season on the years of life lost from respiratory diseases in the elderly related to ambient PM2.5 exposure in Ningbo, China
Source: Environ Health Prev Med. 2021 Jul 17;26:74. doi: 10.1186/s12199-021-00994-6 (PMC8286574; doi:10.1186/s12199-021-00994-6)
Supplement: Supplementary file 1 — Additional file 1: Table S1. WHO standard life table for years of life lost. Table S2. Spearman correlations between air pollutants and meteorological conditions in Ningbo, China, from 2013 to 2016. Table S3. Associations between per 10 μg/m3 increase in PM2.5 and excess risk of respiratory diseases mortality. [file 12199_2021_994_MOESM1_ESM.docx]

**Supplementary Materials**

**Table S1.** WHO standard life table for years of life lost.

| Age | SEYLL | Age | SEYLL | Age | SEYLL |
| --- | --- | --- | --- | --- | --- |
| 0 | 91.94 | 35 | 57.15 | 70 | 23.15 |
| 1 | 91 | 36 | 56.16 | 71 | 22.23 |
| 2 | 90.01 | 37 | 55.17 | 72 | 21.31 |
| 3 | 89.01 | 38 | 54.18 | 73 | 20.4 |
| 4 | 88.02 | 39 | 53.19 | 74 | 19.51 |
| 5 | 87.02 | 40 | 52.2 | 75 | 18.62 |
| 6 | 86.02 | 41 | 51.21 | 76 | 17.75 |
| 7 | 85.02 | 42 | 50.22 | 77 | 16.89 |
| 8 | 84.02 | 43 | 49.24 | 78 | 16.05 |
| 9 | 83.03 | 44 | 48.25 | 79 | 15.22 |
| 10 | 82.03 | 45 | 47.27 | 80 | 14.41 |
| 11 | 81.03 | 46 | 46.28 | 81 | 13.63 |
| 12 | 80.03 | 47 | 45.3 | 82 | 12.86 |
| 13 | 79.03 | 48 | 44.32 | 83 | 12.11 |
| 14 | 78.04 | 49 | 43.34 | 84 | 11.39 |
| 15 | 77.04 | 50 | 42.36 | 85 | 10.7 |
| 16 | 76.04 | 51 | 41.38 | 86 | 10.03 |
| 17 | 75.04 | 52 | 40.41 | 87 | 9.38 |
| 18 | 74.05 | 53 | 39.43 | 88 | 8.76 |
| 19 | 73.05 | 54 | 38.46 | 89 | 8.16 |
| 20 | 72.06 | 55 | 37.49 | 90 | 7.6 |
| 21 | 71.06 | 56 | 36.52 | 91 | 7.06 |
| 22 | 70.07 | 57 | 35.55 | 92 | 6.55 |
| 23 | 69.07 | 58 | 34.58 | 93 | 6.07 |
| 24 | 68.08 | 59 | 33.62 | 94 | 5.6 |
| 25 | 67.08 | 60 | 32.65 | 95 | 5.13 |
| 26 | 66.09 | 61 | 31.69 | 96 | 4.65 |
| 27 | 65.09 | 62 | 30.73 | 97 | 4.18 |
| 28 | 64.1 | 63 | 29.77 | 98 | 3.7 |
| 29 | 63.11 | 64 | 28.82 | 99 | 3.24 |
| 30 | 62.11 | 65 | 27.86 | 100 | 2.79 |
| 31 | 61.12 | 66 | 26.91 | 101 | 2.36 |
| 32 | 60.13 | 67 | 25.96 | 102 | 1.94 |
| 33 | 59.13 | 68 | 25.02 | 103 | 1.59 |
| 34 | 58.14 | 69 | 24.08 | 104 | 1.28 |
|  |  |  |  | 105 | 1.02 |

Note: SEYLL, standard expected years of life lost (from Global Health Estimates Technical Paper WHO/HIS/HSI/GHE/2013.4).

**Table S2.** Spearman correlations between air pollutants and meteorological conditions in Ningbo, China, from 2013 to 2016.

| **Variables** | **PM2.5** | **Ozone 8h** | **NO2** | **SO2** | **Temperature** | **Relative humidity** |
| --- | --- | --- | --- | --- | --- | --- |
| **PM_2.5_ (μg/m^3^)** | 1.00 |  |  |  |  |  |
| **Ozone 8h (μg/m^3^)** | -0.05^*^ | 1.00 |  |  |  |  |
| **NO2 (μg/m^3^)** | 0.74^**^ | -0.21^**^ | 1.00 |  |  |  |
| **SO2 (μg/m^3^)** | 0.76^**^ | -0.15^**^ | 0.70^**^ | 1.00 |  |  |
| **Temperature (℃)** | -0.44^**^ | 0.40^**^ | -0.54^**^ | -0.48^**^ | 1.00 |  |
| **Relative humidity (%)** | -0.22^**^ | -0.40^**^ | -0.13^**^ | -0.38^**^ | 0.14^**^ | 1.00 |

Note: ^*^p<0.05, ^**^p<0.01

**Table S3.** Associations between per 10 µg/m^3^ increase in PM_2.5_ and excess risk of respiratory diseases mortality.

| **Variables** | **Whole year**  **（95%CI）** | **Spring-born（95%CI）** | **Summer-born（95%CI）** | **Autumn-born（95%CI）** | **Winter-born**  **（95%CI）** |
| --- | --- | --- | --- | --- | --- |
| **Single-pollutant model** | 1.06(0.17, 1.95) ^*^ | 0.08(-1.94, 2.11)^*^ | 1.26(-0.59,3.11) | -0.40(-2.05,1.24)^*^ | 2.91(1.35, 4.48) |
| **Two-pollutant model** | | | | | |
| +NO_2_ | 0.87(-0.33,2.08) ^*^ | -1.87(-4.66, 0.92) ^*^ | 1.07(-1.42, 3.56) | -0.42(-2.65,1.81) ^*^ | 3.52(1.43, 5.62) |
| +SO_2_ | 0.61(-0.47, 1.70) | -0.06(-2.57, 2.45) | 0.39(-1.85, 2.63) | -0.80(-2.83,1.23) ^*^ | 2.53(0.65, 4.41) |
| +O_3_ | 0.70(-0.25, 1.64) | -0.72(-2.89, 1.45) ^*^ | 1.13(-0.84, 3.09) | -0.64(-2.39, 1.11) ^*^ | 2.51(0.85, 4.18) |

Note: Changes were presented at 4-day moving average concentration (mv04). Values are shown as percentage. **p* < 0.05 indicated compared with winter-born.
